# Comparisons of Efficacy of Intravitreal Aflibercept and Ranibizumab in Eyes with Diabetic Macular Edema

**DOI:** 10.1155/2017/1747108

**Published:** 2017-07-03

**Authors:** Norihiro Shimizu, Toshiyuki Oshitari, Tomoaki Tatsumi, Yoko Takatsuna, Miyuki Arai, Eiju Sato, Takayuki Baba, Shuichi Yamamoto

**Affiliations:** Department of Ophthalmology and Visual Science, Chiba University Graduate School of Medicine, Chiba, Japan

## Abstract

We compared the efficacy of intravitreal aflibercept (IVA) to intravitreal ranibizumab (IVR) injections in eyes with diabetic macular edema (DME). The medical records of 49 eyes of 36 patients who were diagnosed with DME and had received IVR and 46 eyes of 40 patients who had received IVA treatment were reviewed. The central macular thickness (CMT) and best-corrected visual acuity (BCVA) were measured at the baseline and at 1, 3, and 6 months after the IVR or IVA. The mean number of injections of IVR was 2.6 ± 1.1 and of IVA was 2.7 ± 1.4. At 6 months, the CMT was significantly thinner than the baseline after IVR and after IVA. The mean BCVA was significantly better than the baseline after IVR only at 1 and 3 months and after IVA at 1 and 6 months. The BCVA of eyes with serous retinal detachment (SRD) was significantly better at 1 month after the IVR and at 1 month and 6 months after the IVA. The BCVAs improved more significantly in the SRD+ group than in the SRD− group. The effects of IVA persist longer than that of IVR. The effectiveness of both IVR and IVA was not dependent on the presence of SRD (IRB#2107).

## 1. Introduction

Diabetic macular edema (DME) is one of the most common causes of moderate vision reduction in patients with diabetic retinopathy [1]. A recent meta-analysis of 22,896 diabetic patients showed that the prevalence of DME was 6.81% [2]. There are several therapies for DME such as focal/grid laser photocoagulation, corticosteroids, subthreshold micropulse diode laser photocoagulation, and pars plana vitrectomy. However, intravitreal injections of vascular endothelial growth factor (VEGF) antibodies have become the gold standard therapy for DME worldwide. Several clinical trials strongly suggest that repeated intravitreal injections of anti-VEGF antibodies significantly improved the visual acuity of patients with DME [3–8]. However, frequent anti-VEGF injections are prohibitive for most patients because of the high costs of the anti-VEGF drugs.

In Japan, ranibizumab and aflibercept have been granted on-label use for the treatment of DME. The drug prices of a single injection of ranibizumab and aflibercept in Japan are ¥157,776 (approx. $1,500) and ¥142,605 (approx. $1,400), respectively. Most patients must pay 30% of the medical costs before each injection in addition to the annual medical insurance fees. Thus, frequent injections of anti-VEGF antibodies are not performed on most patients. In representative studies such as the VISTA and VIVID studies [8], the mean number of injections of aflibercept was 9–12 times/year, and in the REVEAL study [7], the mean number of ranibizumab injections was 7-8 times/year. On the other hand, the mean number of injections of ranibizumab was only 4 for a period of 18 months in the PRIDE study, which is the representative study on a practical protocol for IVR injections [9]. The lower number of injections of ranibizumab indicates that it would be more cost effective, but it has not been determined whether this lower number of ranibizumab injections will be as effective in resolving a DME.

Thus, the purpose of this study was to compare the efficacy of IVR and IVA based on a practical protocol in eyes with DME.

## 2. Patients and Methods

The medical records of 49 eyes of 36 patients who were diagnosed with DME and had received IVR treatment in the Chiba University Hospital from March to December in 2014, and 46 eyes of 40 patients who were diagnosed with DME and had received IVA treatment from December in 2014 to October in 2015 were reviewed. DME patients with a central macular thickness (CMT) > 250 *μ*m were studied. Eyes with a CMT < 250 *μ*m, an epiretinal membrane, or vitreomacular traction were excluded. Patients with prior brain ischemia or ischemic heart diseases were also excluded. The injection protocol was 1–3 times consecutive monthly administration, and if the CMT was >300 *μ*m, another injection was given. However, if the patients did not agree to the injection, vitrectomy was performed in these cases of refractory DME.

All of the procedures conformed to the tenets of the World Medical Association Declaration of Helsinki. A written Informed consent was obtained from all patients and approval for this study was obtained from the Institutional Review Board of the Graduates School of Medicine, Chiba University, Japan (number 2107).

The best-corrected visual acuity (BCVA) and the CMT were measured before and at 1, 3, and 6 months after the IVR and IVA. The eyes with DMEs were classified as the serous retinal detachment (SRD) type or not the SRD type by the optical coherence tomographic images (SD-OCT, Heidelberg Engineering, Heidelberg, Germany). A SRD was present (SRD+) in 19 eyes in the IVR group and in 11 eyes in the IVA group and not present (SRD−) in 30 eyes in the IVR group and 35 eyes in the IVA group.

The clinical data and demographics of the patients before the IVR or IVA injections are presented in Table 1. Except for the CMTs, all parameters including age, sex, HbA1c, BCVA, number of SRD types, and injection times were not significantly different in the two groups. In the IVR group, 37 eyes were previously treated with sub-Tenon's capsule triamcinolone acetonide (STTA) injections, 26 eyes had photocoagulation for microaneurysms, 16 eyes had panretinal photocoagulation (PRP), and 2 eyes had pars plana vitrectomy. In the IVA group, 36 eyes were previously treated with STTA, 28 eyes had photocoagulation for microaneurysms, 30 eyes had PRP, and 25 eyes had other types of anti-VEGF antibody injections including 24 eyes with ranibizumab and 1 eye with bevacizumab. The mean interval between injections was 2.6 ± 1.1 months for IVR and 2.7 ± 1.4 months for IVR (*P* = 0.507, Student's *t*-tests). The mean duration from the development of DME to the first IVR was 14.0 ± 10.7 months, and that from the development of DME to the first IVA was 19.3 ± 16.5 months. In the IVA group, 24 eyes were received IVR before IVA. Twenty-two eyes were received IVA without IVR. After November 2014, all patients with persistent or recurrent DME treated with IVR were treated with IVA because IVA becomes a first choice of the medical treatment for DME in our hospital (see Supplemental Figure in Supplementary Material available online at https://doi.org/10.1155/2017/1747108). The changing of medical treatment protocols of a first choice of therapy for DME in our hospital is shown in Supplemental Figure as a reference.

The data are presented as the means ± standard deviations or standard errors. The significance of differences in the data was determined by Student's* t*-tests, paired* t*-tests, chi-square tests, one-way analyses of variance (ANOVA), and repeated measured ANOVA. A *P* < 0.05 was considered significant.

## 3. Results

**Figure 1:**
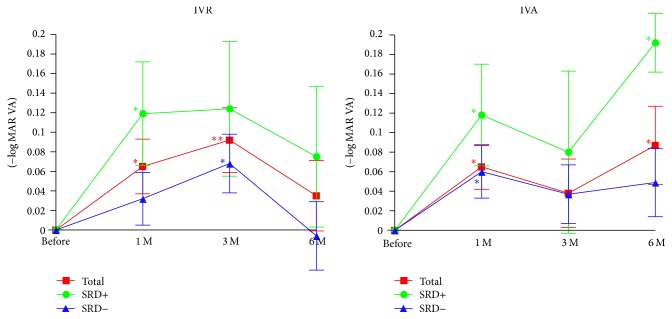
Changes in the mean best-corrected visual acuity (BCVA) expressed in logarithm of the minimum angle of resolution (logMAR) units before and after IVA and IVR treatment in eyes with (SRD+) and without (SRD−) a serous retinal detachment. After 6 months of IVA treatment, the BCVA is significantly improved from the baseline, but after 6 months after IVR, the effectiveness of improvement of BCVA does not persist. Data are expressed as the mean ± SEM. ^*∗*^*P* < 0.05; ^*∗∗*^*P* < 0.01 relative to the baseline of the BCVA. SRD, serous retinal detachment; IVR, intravitreal ranibizumab injection; IVA, intravitreal aflibercept injection.

The BCVAs before and after the IVR and IVA injections are shown in Figure 1 and Table 2. The BCVAs were significantly better than the baseline BCVAs at 1 and 3 months after the IVR (*P* = 0.0175 and *P* = 0.0077, resp.; Figure 1) but not significantly better at 6 months after the IVR. On the other hand, the BCVAs were significantly better at 1 and 6 months after the IVA (*P* = 0.0108 and *P* = 0.0413, resp.; Figure 1). In the SRD+ group, the BCVA was significantly improved only at 1 month after the IVR (*P* = 0.0379; Figure 1). In the SRD− group of IVR, the BCVA was significantly improved only at 3 months after the IVR (*P* = 0.0380; Figure 1). On the other hand, in the SRD+ group, the BCVAs were significantly improved at 1 and 6 months after the IVA (*P* = 0.0456 and *P* = 0.0307, resp.; Figure 1). In the SRD− group, the BCVA was significantly improved only at 1 month after IVA (*P* = 0.0175; Figure 1).

Repeated measured ANOVA showed a significant difference in the BCVA between the SRD+ and SRD− groups (*P* = 0.0443). Thus, the BCVAs improved more significantly in the SRD+ group than in the SRD− group after both IVR and IVA.

**Figure 2:**
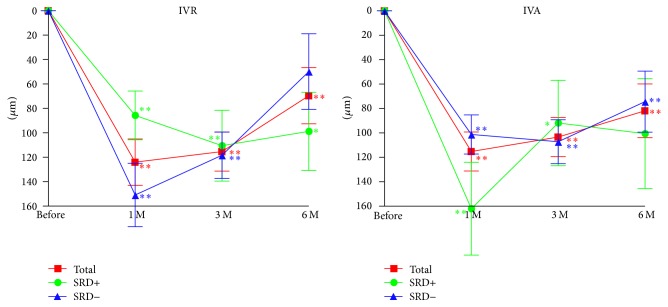
Changes of the mean CMT before and after IVA and IVR treatment in eyes with or without a SRD. The CMT thickness is still significantly reduced 6 months after both IVA and IVR. Data are expressed as mean ± SEM. ^*∗*^*P* < 0.05; ^*∗∗*^*P* < 0.01 relative to the baseline of the CMT. CMT, central macular thickness; SRD, serous retinal detachment; IVR, intravitreal ranibizumab injection; IVA, intravitreal aflibercept injection.

The CMTs before and after IVR and IVA treatments are shown in Figure 2 and Table 2. The mean CMT was significantly reduced at 1, 3, and 6 months after both IVR (*P* < 0.0001, *P* < 0.0001, and *P* = 0.0053, resp.; Figure 2) and the IVA treatment (*P* < 0.0001; Figure 2). In the SRD+ group, the mean CMTs was significantly reduced only at 1 and 3 months after IVR (*P* = 0.0004 and *P* = 0.0012, resp.; Figure 2). In the SRD− group, the mean CMT was reduced at 1, 3, and 6 months after the IVR (*P* < 0.0001, *P* < 0.0001, and *P* = 0.0313, resp.; Figure 2). In the SRD− group, the mean CMT was significantly reduced at 1, 3, and 6 months after the IVA (*P* < 0.0001, *P* < 0.0001, and *P* = 0.0003, resp.; Figure 2). In the SRD+ group, the mean CMT was significantly reduced at 1 and 3 months after IVA (*P* < 0.0010 and *P* = 0.0290, resp.; Figure 2).

**Figure 3:**
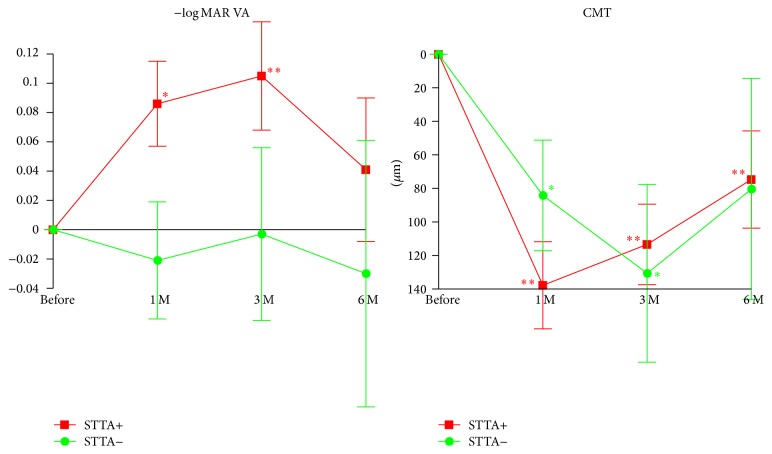
Changes in the mean BCVA (logMAR units) and CMT relative to the baseline with and without a previous STTA treatment. In eyes refractory to STTA, the IVR significantly improves the BCVA at 3 and 6 months after the IVR treatment. STTA, sub-Tenon's triamcinolone acetonide; CMT, central macular thickness; Data are expressed as mean ± SEM. ^*∗*^*P* < 0.05; ^*∗∗*^*P* < 0.01 relative to the baseline of the BCVA and the CMT.

Before the on-label use of IVR was permitted, STTA was the first choice medical treatment for DME in our hospital [10–12]. Thus, we were able to examine the efficacy of IVR on eyes with DME that had not responded to the STTA treatment (Figure 3 and Table 3). The average intervals of the last STTA and the first IVR was 7.3 ± 5.9 months. In eyes with the previous STTA treatment, the BCVAs were significantly improved at 1 and 3 months after the IVR (*P* = 0.0106 and *P* = 0.0079, resp.; Figure 3). In eyes without previous STTA treatment, the BCVAs were not significantly improved at any time after the IVR.

The STTA+ group had better BCVAs after IVR than the STTA− group; one-way ANOVA showed a significant difference between the STTA+ group and the STTA− group (*P* = 0.0439).

In eyes with the previous STTA treatment, the CMTs were significantly reduced at 1, 3, and 6 months after IVR (*P* < 0.0001, *P* < 0.0001, and *P* = 0.030; Figure 3). In eyes without the previous STTA treatment, the CMTs were significantly thinner at 1 and 3 months after the IVR (*P* = 0.0305 and *P* = 0.0180, resp.; Figure 3).

**Figure 4:**
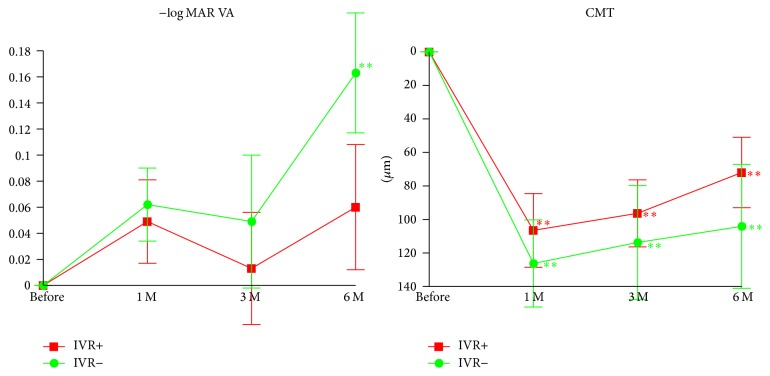
Changes in the mean BCVA (logMAR units) and CMT relative to the baseline in eyes with or without the previous IVR treatment. In eyes refractory to IVR, IVA significantly reduced CMT at 6 months after IVA treatment but did not significantly improved VA at any time after IVA treatment. IVR, intravitreal ranibizumab injection; CMT, central macular thickness. ^*∗∗*^*P* < 0.01 relative to the baseline of the BCVA and the CMT. Data are expressed as mean ± SEM.

Nine months after the use of IVR was permitted, IVA was granted its use in eyes with DME in Japan. We then completely shifted the first choice treatment for DME from IVR into IVA. Thus, we have examined the effectiveness of IVA on eyes with DME that were refractory to IVR treatment (Figure 4 and Table 4). In eyes with the previous IVR treatment, the mean BCVA was not significantly improved at any time after the IVA. However, in eyes without previous IVR treatment, the BCVA was significantly improved at 6 months after the IVA (*P* = 0.0063; Figure 4). In eyes with and without previous IVR treatment, the mean CMT was significantly reduced at all times (at 1, 3, and 6 months) after the IVA (*P* < 0.0001, *P* = 0.0021, and *P* = 0.0053, resp.; Figure 4).

## 4. Discussion

The results of recent clinical trials have indicated that eyes with poorer baseline BCVAs had significantly better BCVAs at 2-years after IVA than after intravitreal bevacizumab (IVB) injections [13, 14]. However, the BCVAs were not significantly different from that after IVR at 2-years [13, 14]. The mean number of injections was 9 in the aflibercept group, 10 in the bevacizumab group, and 10 in the ranibizumab group for the first year [13]. Because the participants did not pay for the ranibizumab and aflibercept, the number of injections was higher than in our study, namely, approximately 3 times more for the 6-month experimental period. In the PRIDE study, which is a representative study on a practical protocol for IVR injections, the frequency of IVR was 4/18 months [9]. Our results indicated that the improvement of the BCVA after IVA was maintained for 6 months which was significantly longer than that after IVR because the improvement of the BCVA after IVR was maintained for only 3 months.

Because the injection numbers of this study are fewer than other clinical trials and most patients have recurrent or persistent DME, it is difficult to improve BCVAs significantly after 6 months of IVR. However, aflibercept is designed as fusion proteins with Fc domain of human immunoglobulin G1 and VEGF receptors 1 and 2. Thus, the binding affinity of VEGF-A is 100 times greater than ranibizumab. In addition, only aflibercept can bind to placental growth factor. Therefore, even in the fewer injection numbers, IVA may show longer lasting effects in the improvement of BCVA 6 months after injection in this study.

Because of the fewer number of injections and longer lasting effects in the improvement of the BCVA after IVA, we recommend IVA for DME. However, a recent study reported that the cost-effectiveness of aflibercept and ranibizumab for DME is poorer than that of bevacizumab [15]. In cases of refractory DME, frequent anti-VEGF antibodies injection may be required. Thus, it may be necessary to combine other therapies such as subthreshold photocoagulation to reduce the number of injection times [15].

We had a chance of examining the effectiveness of IVR on eyes that were refractory to STTA treatment. The results indicated that the effectiveness of IVR was better in eyes with previous STTA treatment than in eyes without the previous STTA treatment. This may be because several cytokines such as IL-6 or MCP-1 are involved in the development of DME [16, 17], and these cytokines cannot be decreased by anti-VEGF antibody [18]. However, steroids can reduce the inflammatory cytokines other than VEGF [19]. Our results showed that VEGF may be involved in the recurrence of DMEs, and the effectiveness of IVR in eyes with previous STTA treatment is better than in eyes without the previous STTA treatment because of the reduction of other cytokines after triamcinolone injection [18, 20]. Thus, anti-VEGF antibody injections including IVR can be a therapeutic option for eyes with DME which are refractory to STTA.

Nine months after the approval of IVR, IVA was approved for on-label use for DME treatment in Japan. Thus, we had a chance to examine the effectiveness of IVA on DME eyes that were refractory to IVR treatment. Our findings suggested that IVA may be effective in reducing the CMT in DM eyes refractory to IVR. However, IVA did not improve the BCVA in eyes refractory to IVR injection. Cytokines other than VEGF may be involved in these eyes with DME refractory to IVR [16–18, 20]. In such eyes, STTA or subthreshold photocoagulation combined with IVA should be considered.

Our previous study showed that STTA was not more effective for the SRD+ type of DME compared to the SRD− type of DME [12]. Thus, we have examined the efficacy of IVR or IVA in eyes with and without a SRD. Our findings suggested that the effectiveness of IVR and IVA is significantly better in the presence of SRD than in the absence of SRD. Thus, IVR and IVA can be therapeutic options for the treatment of DME eyes with and without SRD.

The exact mechanism(s) associated with the development of SRD has not been determined. Steroids downregulate VEGF expression [19] and might be considered to treat DME although it may be difficult to reduce the accumulated VEGF. Because both types of anti-VEGF were effective in resolving the SRDs, VEGF may be accumulated in the fluid of the SRD. We suggest that the anti-VEGF antibodies may reduce the activity of the accumulated VEGF in the subretinal space.

This study has several limitations. First, this was a retrospective study on small numbers of eyes. In addition, the duration of this study was short. Thus, further studies on a larger number of patients and for a longer period are needed to compare the efficacy of IVR and IVA in eyes with DME.

Second, the demographics of the patients the IVR and IVA groups were different because of the retrospective nature of this study. Thus, the conclusion of this study should be interpreted with caution.

In conclusion, the duration of the effects of IVR and IVA is different for the CMT and BCVA, but the effectiveness of IVA in improving the BCVA may be better than IVR. The effectiveness of IVR and IVA is not dependent on the presence or absence of a SRD. We conclude that IVR can be effective in reducing the CMT in eyes with DME refractory to STTA and that IVA may be effective in reducing CMT in DM eyes refractory to IVR. Thus, we recommend IVA treatments for eyes with DME.

## Supplementary Material

Supplemental Figure: The schema of the changing of the medical treatment protocols.

## Figures and Tables

**Table 1:** Clinical data and features.

	IVA group	IVR group	*P* value
Number of eyes	46	49	
Age (years)	64.5 ± 10.7	62.6 ± 10.0	0.239
HbA1c (%)	8.0 ± 1.8	7.86 ± 2.0	0.735
sex (men : women)	20 : 20	22 : 14	0.331
BCVA (logMAR units before)	0.39 ± 0.29	0.48 ± 0.31	0.716
CMT (*μ*m; before)	482 ± 106	536 ± 141	0.039
Injection times	2.7 ± 1.4	2.6 ± 1.1	0.507
SRD (SRD+ : SRD−)	11 : 35	19 : 30	0.119

BCVA, best-corrected visual acuity; CMT, central macular thickness; SRD, serous retinal detachment; IVA, intravitreal aflibercept injection; IVR, intravitreal ranibizumab injection.

**Table 2:** The real values of BCVA (logMAR VA) and CMT before and after IVR and IVA treatment in eyes with (SRD+) and without (SRD−) a serous retinal detachment.

	BCVA (logMAR units)	CMT (*μ*m)
Before IVR (total)	0.48 ± 0.31	536 ± 141
1 M after IVR	0.41 ± 0.30	412 ± 104
3 M after IVR	0.39 ± 0.32	421 ± 149
6 M after IVR	0.45 ± 0.33	466 ± 177
Before IVA (total)	0.39 ± 0.29	482 ± 106
1 M after IVA	0.33 ± 0.31	367 ± 96
3 M after IVA	0.35 ± 0.35	379 ± 116
6 M after IVA	0.30 ± 0.33	400 ± 120

Before IVR (SRD+)	0.46 ± 0.29	496 ± 133
1 M after IVR	0.34 ± 0.28	410 ± 128
3 M after IVR	0.33 ± 0.30	386 ± 126
6 M after IVR	0.37 ± 0.30	397 ± 157
Before IVR (SRD−)	0.48 ± 0.34	550 ± 142
1 M after IVR	0.45 ± 0.31	401 ± 93
3 M after IVR	0.42 ± 0.33	432 ± 158
6 M after IVR	0.49 ± 0.36	500 ± 177

Before IVA (SRD+)	0.51 ± 0.25	512 ± 99
1 M after IVA	0.39 ± 0.26	349 ± 162
3 M after IVA	0.43 ± 0.28	420 ± 141
6 M after IVA	0.32 ± 0.31	411 ± 147
Before IVA (SRD−)	0.37 ± 0.3	475 ± 109
1 M after IVA	0.31 ± 0.32	374 ± 96
3 M after IVA	0.33 ± 0.37	368 ± 105
6 M after IVA	0.32 ± 0.34	401 ± 111

**Table 3:** The real values of BCVA (logMAR VA) and CMT in eyes with DME treated with IVR with and without a previous STTA treatment.

	BCVA (logMAR units)	CMT (*μ*m)
STTA+ before IVR	0.48 ± 0.31	540 ± 143
STTA+ 1 M after IVR	0.40 ± 0.28	403 ± 109
STTA+ 3 M after IVR	0.38 ± 0.28	426 ± 27
STTA+ 6 M after IVR	0.43 ± 0.31	464 ± 171

STTA− before IVR	0.42 ± 0.36	493 ± 134
STTA− 1 M after IVR	0.45 ± 0.39	409 ± 108
STTA− 3 M after IVR	0.43 ± 0.45	363 ± 90
STTA− 6 M after IVR	0.45 ± 0.45	413 ± 187

**Table 4:** The real values of BCVA (logMAR VA) and CMT in eyes with DME treated with IVA with or without the previous IVR treatment.

	BCVA (logMAR units)	CMT (*μ*m)
IVR+ before IVA	0.42 ± 0.32	486 ± 118
IVR+ 1 M after IVA	0.37 ± 0.34	390 ± 106
IVR+ 3 M after IVA	0.41 ± 0.35	384 ± 124
IVR+ 6 M after IVA	0.35 ± 0.35	414 ± 133

IVR− before IVA	0.35 ± 0.26	471 ± 87
IVR− 1 M after IVA	0.29 ± 0.26	345 ± 54
IVR− 3 M after IVA	0.30 ± 0.35	357 ± 99
IVR− 6 M after IVA	0.19 ± 0.28	367 ± 133
