# Supplementary material for: Comparisons of Efficacy of Intravitreal Aflibercept and Ranibizumab in Eyes with Diabetic Macular Edema
Source: Biomed Res Int. 2017 Jul 3;2017:1747108. doi: 10.1155/2017/1747108 (PMC5512051; doi:10.1155/2017/1747108)

**Supplemental Figure**. **The schema of the changing of the medical treatment protocols**. The first choice of the medical treatment for diabetic macular edema (DME) has been changed in our hospital. Sub-Tenon’s capsule triamcinolone acetonide (STTA) injection was a first choice of the medical treatment for DME before the on-label use of intravitreal ranibizumab (IVR) injection was permitted. Since February 2014, IVR has been a first choice of the medical treatment. After November 2014, intravitreal aflibercept (IVA) injection becomes a first choice of the medical treatment for DME. Thus, we compared the efficacy of IVA to IVR after 6M of injections in eyes with DME. In addition, we were able to examine the efficacy of IVR on eyes with DME that had not responded to the STTA treatment. Furthermore, we had a chance to examine the effectiveness of IVA on DME eyes that were refractory to IVR treatment.


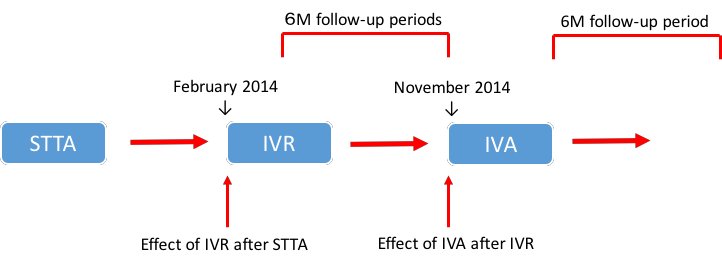

Supplement: Supplementary file 1 — Supplemental Figure: The schema of the changing of the medical treatment protocols. [file 1747108.f1.docx]
